# Supplementary material for: Fatty acid analogue N-arachidonoyl taurine restores function of IKs channels with diverse long QT mutations
Source: eLife. 2016 Sep 30;5:e20272. doi: 10.7554/eLife.20272 (PMC5081249; doi:10.7554/eLife.20272)
Supplement: Supplementary file 1. — DOI: http://dx.doi.org/10.7554/eLife.20272.020 [file elife-20272-supp1.docx]

|  | **-KCNE1** | | | | |  | **+KCNE1** | | | | | |  |
| --- | --- | --- | --- | --- | --- | --- | --- | --- | --- | --- | --- | --- | --- |
|  | ***s***  **(mV)** | ***V*_50_**  **(mV)** | **T_50,open_**  **(ms)** | **T_50,close_**  **(ms)** | ***n*** |  | ***s***  **(mV)** | ***V*_50_**  **(mV)** | **T_50,open_**  **(ms)** | **T_50,close_**  **(ms)** | | ***n*** | |
| Kv7.1 (WT) | 09.3 ± 0.4 | −29.4 ± 1.4 | 048 ± 5.7 | 977 ± 59 | 6 |  | 14.0 ± 1.2 | +20.1 ± 3.2 | 1980 ± 710 | 967 ± 470 | 5 | |  |
|  |  |  |  |  |  |  |  |  |  |  |  | |  |
| Kv7.1/F193L | 10.8 ± 0.4 | −29.6 ± 0.4 | <25 | nd | 7 |  | 12.3 ± 0.6 | +23.1 ± 1.1 | 1455 ± 760 | 483 ± 210 | 11 | |  |
| Kv7.1/V215M | 09.6 ± 0.2 | −25.2 ± 1.4 | 046 ± 5 | 499 ± 51 | 5 |  | 11.3 ± 0.4 | +47.5 ± 1.8 | 1755 ± 124 | 251 ± 120 | 8 | |  |
| Kv7.1/S225L | 13.8 ± 0.9 | +13.8 ± 1.5 | 269 ± 31 | 644 ± 57 | 8 |  | 13.6 ± 0.7 | +51.3 ± 2.7 | 2297 ± 697 | 458 ± 490 | 7 | |  |
| Kv7.1/L251A | 08.0 ± 0.5 | 0−3.2 ± 1.0 | 876 ± 102 | 3464 ± 157 | 6 |  | 16.3 ± 0.8 | +34.8 ± 8.3 | 2015 ± 224 | 766 ± 510 | 5 | |  |
| Kv7.1/F351A | 10.0 ± 0.8 | +34.7 ± 2.3 | 1113 ± 62 | 543 ± 40 | 12 |  | nd | > +50 | nd | nd | 8 | |  |
| Kv7.1/R583C | 13.4 ± 1.1 | −42.8 ± 2.3 | nd | nd | 7 |  | 15.8 ± 1.4 | +42.4 ± 3.2 | 1814 ± 124 | 588 ± 110 | 8 | |  |
|  |  |  |  |  |  |  |  |  |  |  |  | |  |
| Kv7.1 + E1/K70N | N/A | N/A | N/A | N/A |  |  | 11.7 ± 0.3 | +43.0 ± 1.5 | 0913 ± 102 | 166 ± 800 | 7 | |  |
| Kv7.1 + E1/S74L | N/A | N/A | N/A | N/A |  |  | 10.4 ± 0.6 | +41.8 ± 2.2 | 1784 ± 102 | 286 ± 700 | 8 | |  |

*s* is the slope factor and *V*_50_ the midpoint of the *G*(*V*) curve estimated from the Boltzmann fits (see Methods for details).

For –KCNE1: T_50,open_ is the time it takes to reach 50% of the current in the end of a 3 s long test pulse to +40 mV. T_50,close_ is the time it takes to reduce the amplitude (= instantaneous tail current – steady state tail current in the end of a 5 s pulse) of the tail current by 50% when stepping to a tail pulse to −20.

For +KCNE1: T_50,open_ is the time it takes to reach 50% of the current in the end of a 5 s long test pulse to +40 mV. T_50,close_ is the time it takes to reduce the amplitude (= instantaneous tail current – steady state tail current in the end of a 5 s pulse) of the tail current by 50% when stepping to a tail pulse to −20.

T_50,open_ for F193L is an approximation due to very fast activation kinetics. T_50,close_ for F193L is not determined due to lack of closing at −20 mV. T_50,open_ and T_50,close_ is not determined for R583C due to pronounced inactivation at voltages higher than 0 mV. *V*_50_ for F351A+KCNE1 could not be determined due to the much shifted *G*(*V*) of this mutant. *V*_50_ for F351A+KCNE1 is more positive than the *V*_50_ for S225L+KCNE1 and thus +50 mV is given as a lowest estimate of the *V*_50_. The current at +40 mV for F351A+KCNE1 was too small to analyze current amplitude and kinetics. nd = not determined. N/A = not applicable. Mean ± SEM.
