# Supplementary material for: Fatty acid analogue N-arachidonoyl taurine restores function of IKs channels with diverse long QT mutations
Source: eLife. 2016 Sep 30;5:e20272. doi: 10.7554/eLife.20272 (PMC5081249; doi:10.7554/eLife.20272)
Supplement: Supplementary file 2. — DOI: http://dx.doi.org/10.7554/eLife.20272.021 [file elife-20272-supp2.docx]

|  |  | | | | |  | **+Kv7.1^wt^ or KCNE1^wt^** | | | | |
| --- | --- | --- | --- | --- | --- | --- | --- | --- | --- | --- | --- |
|  | ***s***  **(mV)** | ***V*_50_**  **(mV)** | **T_50,open_**  **(ms)** | **T_50,close_**  **(ms)** | ***n*** |  | ***s***  **(mV)** | ***V*_50_**  **(mV)** | **T_50,open_**  **(ms)** | **T_50,close_**  **(ms)** | ***n*** |
| Kv7.1 (WT) +E1 | 14.0 ± 1.2 | +20.1 ± 3.2 | 1980 ± 71 | 967 ± 47 | 5 |  | N/A | N/A | N/A | N/A |  |
|  |  |  |  |  |  |  |  |  |  |  |  |
| Kv7.1/F193L +E1 | 12.3 ± 0.6 | +23.1 ± 1.1 | 1455 ± 76 | 483 ± 21 | 11 |  | 13.5 ± 0.3 | +29.5 ± 2.9 | 2082 ± 97 | 732 ± 51 | 8 |
| Kv7.1/V215M +E1 | 11.3 ± 0.4 | +47.5 ± 1.8 | 1755 ± 124 | 251 ± 12 | 8 |  | 13.1 ± 0.7 | +36.6 ± 1.6 | 2015 ± 53 | 512 ± 26 | 10 |
| Kv7.1/S225L +E1 | 13.6 ± 0.7 | +51.3 ± 2.7 | 2297 ± 697 | 458 ± 49 | 7 |  | 13.3 ± 0.6 | +35.0 ± 1.5 | 2191 ± 66 | 513 ± 51 | 8 |
| Kv7.1/L251A +E1 | 16.3 ± 0.8 | +34.8 ± 8.3 | 2015 ± 224 | 766 ± 51 | 5 |  | 13.3 ± 0.7 | +14.3 ± 3.8 | 1525 ± 203 | 650 ± 121 | 7 |
| Kv7.1/F351A +E1 | nd | > +50 | nd | nd | 8 |  | 15.3 ± 1.1 | +29.1 ± 5.2 | 1645 ± 68 | 572 ± 72 | 7 |
| Kv7.1/R583C +E1 | 15.8 ± 1.4 | +42.4 ± 3.2 | 1814 ± 124 | 588 ± 110 | 8 |  | 15.6 ± 0.6 | +44.6 ± 4.6 | 2414 ± 35 | 1073 ± 68 | 7 |
|  |  |  |  |  |  |  |  |  |  |  |  |
| Kv7.1 + E1/K70N | 11.7 ± 0.3 | +43.0 ± 1.5 | 913 ± 102 | 166 ± 8 | 7 |  | 14.0 ± 0.7 | +25.5 ± 2.2 | 1659 ± 148 | 609 ± 49 | 13 |
| Kv7.1 + E1/S74L | 10.4 ± 0.6 | +41.8 ± 2.2 | 1784 ± 102 | 286 ± 7 | 8 |  | 14.0 ± 0.6 | +20.6 ± 4.6 | 1689 ± 141 | 517 ± 32 | 8 |

*s*, *V*_50_, T_50,open_ and T_50,close_ are defined as in Supplementary File 1. The current at +40 mV for F351A+KCNE1 was too small to analyze current amplitude and kinetics. nd = not determined. E1 = KCNE1. N/A = not applicable. Mean ± SEM.
