# Supplementary material for: Fatty acid analogue N-arachidonoyl taurine restores function of IKs channels with diverse long QT mutations
Source: eLife. 2016 Sep 30;5:e20272. doi: 10.7554/eLife.20272 (PMC5081249; doi:10.7554/eLife.20272)
Supplement: Supplementary file 3. — DOI: http://dx.doi.org/10.7554/eLife.20272.022 [file elife-20272-supp3.docx]

|  |  |  | | | | | |  | | **+Kv7.1^wt^ or KCNE1^wt^** | | | | | |  |
| --- | --- | --- | --- | --- | --- | --- | --- | --- | --- | --- | --- | --- | --- | --- | --- | --- |
|  | **Δ*V*_50_**  **(mV)** | **ΔΔ*G*_o_**  **(kJ/mol)** | **A_N-AT_/**  **A_Ctrl_** | **T_50,openN-AT/_**  **T_50,openCtrl_** | **T_50,closeN-AT/_**  **T_50,closeCtrl_** | ***n*** |  | | **Δ*V*_50_**  **(mV)** | | **ΔΔ*G*_o_**  **(kJ/mol)** | **A_N-AT_/**  **A_Ctrl_** | **T_50,openN-AT/_**  **T_50,openCtrl_** | **T_50,closeN-AT/_**  **T_50,closeCtrl_** | ***n*** | |
| Kv7.1 (WT) +E1 | –27.0 ± 2.5 | –4.9 ± 0.7 | 1.9 ± 0.3 | 0.6 ± 0.0 | 1.2 ± 0.1 | 5 |  | | N/A | | N/A | N/A | N/A | N/A |  | |
|  |  |  |  |  |  |  |  | |  | |  |  |  |  |  | |
| Kv7.1/F193L +E1 | –34.7 ± 2.0 | –6.9 ± 0.5 | 2.2 ± 0.3 | 0.4 ± 0.0 | 1.1 ± 0.0 | 11 |  | | –37.5 ± 5.9 | | –6.8 ± 1.1 | 2.3 ± 0.6 | 0.6 ± 0.1 | 1.0 ± 0.0 | 8 | |
| Kv7.1/V215M +E1 | –39.1 ± 2.1 | –8.4 ± 0.4 | 9.3 ± 2.5 | 0.6 ± 0.0 | 1.2 ± 0.1 | 8 |  | | –34.6 ± 2.0 | | –6.7 ± 0.5 | 3.2 ± 0.7 | 0.4 ± 0.0 | 1.3 ± 0.0 | 9 | |
| Kv7.1/S225L +E1 | –49.4 ± 3.6 | –9.0 ± 1.1 | 5.4 ± 1.3 | 0.6 ± 0.0 | 1.2 ± 0.1 | 6 |  | | –44.3 ± 1.4 | | –8.2 ± 0.4 | 4.0 ± 0.4 | 0.5 ± 0.0 | 1.2 ± 0.1 | 8 | |
| Kv7.1/L251A +E1 | –43.3 ± 6.0 | –6.6 ± 1.2 | 3.7 ± 0.5 | 0.6 ± 0.1 | 1.1 ± 0.2 | 5 |  | | –28.6 ± 2.0 | | –5.3 ± 0.5 | 1.5 ± 0.2 | 0.6 ± 0.1 | 1.1 ± 0.2 | 7 | |
| Kv7.1/F351A +E1 | ~ –30 | nd | nd | nd | nd | 6 |  | | –41.2 ± 4.4 | | –7.1 ± 0.8 | 3.3 ± 0.3 | 0.5 ± 0.1 | 1.2 ± 0.1 | 6 | |
| Kv7.1/R583C +E1 | –33.2 ± 3.3 | –5.4 ± 0.7 | 2.1 ± 0.5 | 0.8 ± 0.1 | 1.5 ± 0.2 | 8 |  | | –36.5 ± 1.8 | | –5.7 ± 0.4 | 4.3 ± 1.3 | 0.7 ± 0.1 | 0.9 ± 0.1 | 7 | |
|  |  |  |  |  |  |  |  | |  | |  |  |  |  |  | |
| Kv7.1 + E1/K70N | –28.6 ± 0.9 | –5.9 ± 0.3 | 3.1 ± 0.2 | 0.7 ± 0.1 | 1.6 ± 0.1 | 7 |  | | –34.9 ± 2.2 | | –6.4 ± 0.5 | 2.6 ± 0.5 | 0.6 ± 0.1 | 1.4 ± 0.2 | 12 | |
| Kv7.1 + E1/S74L | –35.2 ± 2.9 | –8.2 ± 0.7 | 7.5 ± 2.5 | 0.4 ± 0.0 | 1.5 ± 0.0 | 8 |  | | –32.1 ± 4.2 | | –5.7 ± 0.7 | 1.4 ± 0.3 | 0.7 ± 0.1 | 1.2 ± 0.1 | 6 | |

A_N-AT_/A_Ctrl_ is the ratio between the current amplitude for N-AT and control solution in the end of a test pulse for 5 s to +40 mV. T_50,open_ and T_50,close_ are defined as in Supplementary File 1. The N-AT induced Δ*V*_50_ for F351A+KCNE1 is an approximation due to the much shifted voltage dependence of this mutant. The current at +40 mV for F351A+KCNE1 was too small to analyze current amplitude and kinetics. E1 = KCNE1. nd = not determined. N/A = not applicable. Mean ± SEM.
