# Supplementary material for: Fatty acid analogue N-arachidonoyl taurine restores function of IKs channels with diverse long QT mutations
Source: eLife. 2016 Sep 30;5:e20272. doi: 10.7554/eLife.20272 (PMC5081249; doi:10.7554/eLife.20272)
Supplement: Supplementary file 4. — DOI: http://dx.doi.org/10.7554/eLife.20272.023 [file elife-20272-supp4.docx]

| *Rate* | *WT* | *S225L* | *F351A* |
| --- | --- | --- | --- |
| *k_S4_ (s^-1^)* | 1.0 | 1.0 | 1.0 |
| *z_On S4_ (e_0_)* | 0.29 | 0.29 | 0.29 |
| *z_Off S4_ (e_0_)* | −0.66 | −0.66 | −0.66 |
| *V_50 S4_ (mV)*  *c* | −52.5  0.5 | −2.5  0.5 | −52.5  0.5 |
| *e (s^-1^)*  *d (s^-1^)* | 0.5  12 | 0.5  12 | 0.5  12 |
| *z_On gate_ (e_0_)* | 0.063 | 0.063 | 0.063 |
| *z_Off gate_ (e_0_)* | −0.087 | −0.087 | −0.087 |
| *V_50 gate_ (mV)* | −58.5 | −58.5 | 81.5 |
| L | 2.89 | 2.89 | 2.89 |

**Supplementary File 4a. Parameters for K_V_7.1 model in Figure 3 – figure supplement 1.** The rates and gating charges z associated with each transition for wild-type K_V_7.1+KCNE1 were determined from a gobal fit of data in a previous study (Osteen *et al.*, 2010^25^ and 2012^26^). Rates modified for each mutation are indicated in gray.

α(*V*) *= k_S4_*exp(z_On S4_F(V- V_50 S4_)/RT),*

β(*V*) *= k_S4_*exp(z_Off S4_F(V- V_50 S4_)/RT),*

e_n_(*V*) *= e*(exp(z_On gate_F(V- V_50 gate_)/RT))^n^,*

d_n_(*V*) *= d*(exp(z_Off gate_F(V- V_50 gate_)/RT))^n^,* where n is the number of activated S4s.

α'(*V*) *= c**α(*V*)**exp(z_Off S4_F(V- V_50 S4_)/RT)* and β'(*V*) *= c**β(*V*)**(exp(z_Off gate_F(V- V_50 gate_)/RT))*, to ensure detailed balance. *R*, *T*, and *F* have their usual thermodynamic meaning.

25. Osteen, J.D., Gonzalez, C., Sampson, K.J., Iyer, V., Rebolledo, S., Larsson, H.P. & Kass, R.S. KCNE1 alters the voltage sensor movements necessary to open the KCNQ1 channel gate. *Proc Natl Acad Sci U S A* **107,** 22710-22715 (2010).

26. Osteen, J.D., Barro-Soria, R., Robey, S., Sampson, K.J., Kass, R.S. & Larsson, H.P. Allosteric gating mechanism underlies the flexible gating of KCNQ1 potassium channels. *Proc Natl Acad Sci U S A* **109,** 7103-7108 (2012).

| *Rate* | *WT* | *S225L* | *F351A* |
| --- | --- | --- | --- |
| *k_S4_ (s^-1^)* | 0.464 | 0.464 | 0.464 |
| *z_On S4_ (e_0_)* | 0.44 | 0.44 | 0.44 |
| *z_Off S4_ (e_0_)* | −0.35 | −0.35 | −0.35 |
| *V_50 S4_ (mV)* | −116.4 | −66.4 | −116.4 |
| *k_gate_ (s^-1^)* | 0.203 | 0.203 | 0.203 |
| *z_On gate_ (e_0_)* | 0.44 | 0.44 | 0.44 |
| *z_Off gate_ (e_0_)* | −0.77 | −0.77 | −0.77 |
| *V_50 gate_ (mV)* | −7.6 | −7.6 | +132.4 |

**Supplementary File 4b. Parameters for K_V_7.1+KCNE1 model in Figure 3 – figure supplement 1.** The rates and gating charges z associated with each transition for wild-type K_V_7.1+KCNE1 were determined in a previous study (Barro-Soria *et al.*, 2014^24^). Rates modified for each mutation are indicated in gray. *R*, *T*, and *F* have their usual thermodynamic meaning.

α(*V*) *= k_S4_*exp(z_On S4_F(V- V_50 S4_)/RT),*

β(*V*) *= k_S4_*exp(z_Off S4_F(V- V_50 S4_)/RT),*

γ(*V*) *= k_gate_*exp(z_On gate_F(V- V_50 gate_)/RT),*

δ(*V*) *= k_gate_*exp(z_Off gate_F(V- V_50 gate_)/RT).*

24. Barro-Soria, R., Rebolledo, S., Liin, S.I., Perez, M.E., Sampson, K.J., Kass, R.S. & Larsson, H.P. KCNE1 divides the voltage sensor movement in KCNQ1/KCNE1 channels into two steps. *Nat Commun* **5,** 3750 (2014).
